# Observation of Iso-Symmetric Structural and Lifshitz Transitions in Quasi-One-Dimensional CrNbSe_5_

**DOI:** 10.1021/acs.jpcc.6c01534

**Published:** 2026-04-30

**Authors:** Mingyu Xu, Cheng Peng, Shuyuan Huyan, Wenli Bi, Su-Yang Xu, Sergey L. Bud’ko, Paul C. Canfield, Weiwei Xie

**Affiliations:** † Department of Chemistry, 3078 Michigan State University, East Lansing, Michigan 48824, United States; ‡ 17210 Ames National Laboratory, Ames, Iowa 50011, United States; § Department of Physics and Astronomy, Iowa State University, Ames, Iowa 50011, United States; ∥ SmartState Center for Experimental Nanoscale Physics, Department of Physics and Astronomy, 9968 University of South Carolina, Columbia, South Carolina 29208, United States; ⊥ Department of Chemistry and Chemical Biology, Harvard University, Cambridge, Massachusetts 02138, United States

## Abstract

Chalcogenides-rich transition metal compounds host a rich landscape of emergent quantum phenomena that are intimately governed by their quasi-one-dimensional chemical-bonding frameworks and their response to external perturbations such as pressure. Here, we report a pressure-induced iso-symmetric structural transition in the quasi-one-dimensional compound CrNbSe_5_, in which the electronic ground state is controlled not by symmetry breaking but by a continuous reorganization of local bonding interactions. Applied pressure reversibly tunes CrNbSe_5_ between semiconducting and semimetallic states, enabling access to low- and high-carrier electronic regimes through direct modulation of metal–chalcogen bonding. High-pressure single-crystal X-ray diffraction directly resolves the evolution of Cr–Se and Nb–Se bond distances, coordination polyhedra, and connectivity, revealing a fully reversible semimetal–semiconductor–semimetal transition driven by gradual yet cooperative bond rearrangements within a preserved crystallographic symmetry. In contrast to chemical substitution, which irreversibly alters composition and introduces disorder, pressure acts as a clean, continuous control parameter that reshapes the bonding landscape without disrupting structural symmetry. These results establish CrNbSe_5_ as a model system for electronically driven phase switching via tunable chemical bonding, highlighting iso-symmetric bond reorganization as a powerful design principle for pressure-controlled electronic and spintronic functionalities.

## Graphical abstract

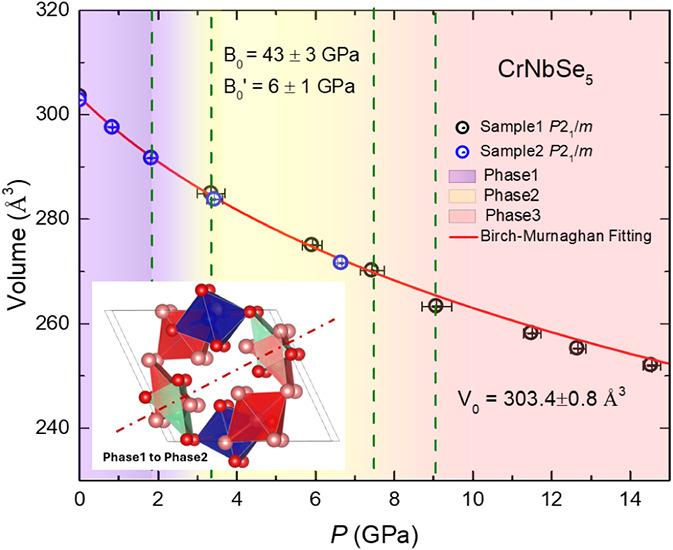

## Introduction

Low-dimensional materials, particularly quasi-one-dimensional (1D) and two-dimensional (2D) van der Waals (vdW) systems,^1–3^ display a rich landscape of emergent electronic, optical, and magnetic properties owing to the reduced spatial dimensionality and enhanced electronic correlations.^4–9^ Among these, the transition metal trichalcogenides (TMTCs, MX_3_; M = Ti, Zr, Hf, V, Nb, Ta; X = S, Se, Te) form a prototypical family of quasi-1D compounds^10–14^ in which strong covalent bonding defines the chain direction, while weaker interchain bonding and van der Waals interactions assemble the chains into sheets and ultimately bulk layered crystals. This unique bonding anisotropy enables TMTCs to combine structural flexibility with highly directional physical behavior, positioning them as promising candidates for next-generation nanoelectronics, optoelectronics, and quantum devices. Historically, TMTCs research has centered on classical CDW-bearing systems such as NbS_3_ and NbSe_3_, where Peierls instabilities, charge density wave (CDW) order, and pressure- or doping-induced superconductivity underscore the delicate interplay between structure, dimensionality, and electronic ground states.^11,15–17^ With the discovery of graphene and the ensuing interest in atomic-scale materials,^3,18^ quasi-1D vdW structures have gained renewed attention, as reductions in dimensionality often enhance electron–electron interactions and enable unconventional quantum phenomena.^1,19^ Indeed, many TMTCs exhibit tunable bandgaps, highly anisotropic electronic dispersion, angle-dependent transport, and strong coupling between structural distortions and electronic instabilities.^10,20^ Pressure, an exceptionally clean tuning parameter, has proven particularly powerful in modulating their electronic phases, as demonstrated by pressure-induced suppression of CDW order, reentrant superconductivity in ZrTe_3_, and structural-electronic reconstructions in layered TaS_2_ and TaSe_2_.^21–24^ These examples highlight the importance of directly probing how local bonding environments evolve under pressure to drive changes in electronic topology and carrier density.

CrNbSe_5_ represents an intriguing extension of the TMTC landscape.^25,26^ Structurally, it can be viewed as a reconstruction of the transition metal dichalcogenides (TMDC) CrSe_2_ and TMTC NbSe_3_, in which the introduction of 3*d* transition metal Cr transforms the NbSe_3_ chain network into duplex-ladder [Cr_2_Nb_2_Se_10_] chains. The combination of potential Cr-based magnetism, quasi-1D chain geometry, and van der Waals layering positions CrNbSe_5_ as a promising system for studying the coupling between chemical bonding, dimensionality, and physical properties. While its parent compounds display pronounced CDW transitions (NbSe_3_) or Peierls-like bond instabilities (CrSe_2_), the structural and electronic behavior of CrNbSe_5_ remains largely unexplored. Notably, its semimetallic ground state suggests the possibility of rich pressure-tuned quantum phenomena, including metal–insulator transitions, Fermi-surface reconstructions, or emergent superconductivity. A fundamental step toward understanding these possibilities is to clarify how the crystal structure and chemical bonding of CrNbSe_5_ evolve under external compression. High pressure can modulate orbital overlaps, tune interchain distances, and alter the relative strengths of Cr–Se and Nb–Se interactions, thereby directly controlling the electronic bandwidth and carrier density. However, such effects cannot be reliably inferred from powder diffraction alone, as quasi-1D materials often exhibit subtle distortions, chain sliding, and bond rearrangements that require single-crystal X-ray diffraction (SCXRD) for accurate determination. SCXRD under pressure provides the unique capability to visualize bond-length variations, coordination evolution, and symmetry-preserving structural changes with atomic precision, the information essential for linking structural tuning to emergent physical properties.

The study of bond rearrangements under various pressures has recently been a focus of extensive study in diverse members of the *AT*_2_ *X*_2_, *AET*_2_ *X*_2_, and *AAET*_4_ *X*_4_ families with *A* = alkali metal, *AE* = alkali-earth or rare-earth, *T* = transition metal, and *X* = pnictogen or chalcogenide. With increasing pressure, the formation of bonds through isostructural phase transitions has led to the creation and/or destruction of magnetic order and moments, remarkable superelastic phenomena, and even superconductivity.^27–35^ These prior studies establish bond rearrangement in isostructural transitions as a powerful and general mechanism for tuning physical properties. CrNbSe_5_ represents a compelling target, as its quasi-one-dimensional bonding framework is expected to be highly susceptible to compression, offering a clean route to induce pronounced changes in its electronic and magnetic ground state.

Here, we combine high-pressure single-crystal X-ray diffraction with complementary physical property measurements to investigate the structural and bonding evolution of CrNbSe_5_. We reveal how pressure drives continuous modifications of the Cr–Se and Nb–Se polyhedra, enabling reversible transitions between distinct electronic states. By correlating these bonding rearrangements with changes in transport behavior, we demonstrate that CrNbSe_5_ is a highly responsive quasi-1D system in which electronic properties can be engineered through subtle pressure-induced structural modulation. This work not only establishes the first comprehensive high-pressure structural study of CrNbSe_5_ but also provides fundamental insight into how chemical bonding controls electronic phase transitions in low-dimensional chalcogenide materials.

## Experimental and Computational Details

### Crystal Synthesis

Single crystals of CrNbSe_5_ were synthesized via a conventional solid-state reaction. Cr pieces (Alfa Aesar, 99% metal basis), Nb slug (Alfa Aesar, 99.95% metal basis, excluding Ta), and Se powder (Thermo Scientific, 99%) were combined in a stoichiometric ratio of 1:1:5 and loaded into an alumina crucible. The crucible was sealed in an evacuated fused-quartz tube under an Ar atmosphere. The sealed tube was heated in a box furnace at a rate of 60 °C/h to 600 °C, held at 600 °C for 3 days, and then furnace-cooled to room temperature. Millimeter-sized single crystals were obtained from the resulting product.

### Ambient Pressure Single-Crystal X-ray Diffraction (SCXRD)

A CrNbSe_5_ single crystal (0.14 × 0.02 × 0.01 mm^3^) was mounted on a nylon loop with Paratone oil and measured at room temperature using a Rigaku XtaLAB Synergy Dualflex diffractometer with a HyPix detector. Data were collected using ω scans with Mo Kα radiation (λ = 0.71073 Å) from a microfocus sealed tube (50 kV, 1 mA). Data collection strategies were optimized using CrysAlisPro (v1.171.43.120a). Data reduction included Lorentz and polarization corrections, numerical absorption correction based on Gaussian integration over a multifaceted crystal model,^36^ and empirical spherical harmonics correction using the SCALE3 ABSPACK algorithm.^37^ Structures were solved and refined using Olex2 with the SHELXTL package.^38–40^

### High-Pressure SCXRD

High-pressure single-crystal XRD was performed at room temperature on the same CrNbSe_5_ crystal characterized at ambient pressure, using a Diacell One20DAC (Almax-easyLab) with 500 μm culet Boehler-Almax anvils and Rigaku XtaLAB Synergy Dualflex diffractometer with a HyPix detector. A stainless-steel gasket (250 μm thick) was preindented to 44 μm, and a 300 μm hole was drilled by the electric discharge machine to hold the sample. A 4:1 methanol–ethanol mixture was used as the pressure-transmitting medium to maintain hydrostatic conditions.^41^ Pressures up to 14.6 GPa were determined using ruby R_1_ fluorescence.^42–44^ Refinement details and atomic coordination parameters for ambient pressure and representative pressures (0.8, 5.9, and 14.6 GPa) are summarized in Tables 1 and 2.

**Table 1:** Crystal Structure and Refinement Data for CrNbSe_5_ at 300 K (Ambient Pressure) and under 0.8, 5.9, and 14.6 GPa^a^

Pressure (GPa)	Ambient	0.8	5.9	14.6
Space Group	*P*2_1_/*m*	*P*2_1_/*m*	*P*2_1_/*m*	*P*2_1_/*m*
Unit Cell dimensions	*a* = 9.1969(9) Å	*a* = 9.1080(18) Å	*a* = 8.7923(13) Å	*a* = 8.4490(18) Å
	*b* = 3.5114(2) Å	*b* = 3.4938(4) Å	*b* = 3.4305(3) Å	*b* = 3.3508(4) Å
	*c* = 10.4164(9) Å	*c* = 10.361(3) Å	*c* = 10.0973(13) Å	*c* = 9.797(2) Å
	β = 115.52(1)°	β = 115.5(3)°	β = 115.44(2)°	β = 114.70(2)°
Volume	303.57(5) Å^3^	297.58(13)	275.02(7)	252.00(9)
*Z*	1	1	1	1
Density (calculated)	5.904	6.023	6.517	7.133
Absorption coefficient	33.508	34.183	36.987	40.365
*F*(000)	470	470	470.0	470
2θ range	7.826 to 84.462	7.958 to 75.22	8.126 to 74.54	8.366 to 74.58
Reflections collected	6972	6174	3415	4260
Independent reflections	2203 [*R*_int_ = 0.0734]	849 [*R*_int_ = 0.2315]	854 [*R*_int_ = 0.0646]	777 [*R*_int_ = 0.3230]
Data/restraints/parameters	2203/0/43	849/0/44	854/0/43	777/0/43
Final *R* indices	R_1_ (*I* > 2σ(*I*)) = 0.0509;	R_1_ (*I* > 2σ(*I*)) = 0.0867;	R_1_ (*I* > 2σ(*I*)) = 0.1466;	R_1_ (*I* > 2σ(*I*)) = 0.1809;
	wR_2_ (*I* > 2σ(*I*)) = 0. 1247	wR_2_ (*I* > 2σ(*I*)) = 0.2190	wR_2_ (*I* > 2σ(*I*)) = 0.3918	wR_2_ (*I* > 2σ(*I*)) = 0.4240
	R_1_ (all) = 0.0765;	R_1_ (all) = 0.2013;	R_1_ (all) = 0.2253;	R_1_ (all) = 0.3004;
	wR_2_ (all) = 0.1330	wR_2_ (all) = 0.3109	wR_2_ (all) = 0.4970	wR_2_ (all) = 0.5211
Largest diff. peak and hole (e^–^/Å^3^)	+4.74/–2.07	+3.02/–4.28	+7.75/–13.24	+6.05/–7.29
Goodness-of-fit on *F* ^2^	1.046	1.010	2.073	1.571

a Values in parentheses represent the estimated standard deviations from the refinements.

**Table 2:** Atomic Coordinates and Equivalent Isotropic Atomic Displacement Parameters (Å^2^) for CrNbSe_5_ at 300 K (Ambient Pressure) and at 300 K under 0.8, 5.9, and 14.6 GPa

P (GPa)	Atom	Wyck.	*x*	*y*	*z*	Occ.	*U*_eq_
**Ambient**	Nb	2*e*	0.77105(8)	1/4	0.86385(7)	1	0.011(2)
	Cr	2*e*	0.06578(14)	1/4	0.40016(14)	1	0.012(2)
	Se1	2*e*	0.46766(9)	1/4	0.25444(8)	1	0.014(15)
	Se2	2*e*	0.33704(9)	1/4	0.00594(8)	1	0.013(15)
	Se3	2*e*	0.72780(9)	1/4	0.58765(8)	1	0.012(14)
	Se4	2*e*	0.15153(10)	1/4	0.65935(9)	1	0.014(15)
	Se5	2*e*	0.01468(9)	1/4	0.13412(7)	1	0.009(13)
**0.8**	Nb	2*e*	0.76870(7)	1/4	0.86350(4)	1	0.028(2)
	Cr	2*e*	0.06550(12)	1/4	0.40050(7)	1	0.022(2)
	Se1	2*e*	0.47120(8)	1/4	0.25460(5)	1	0.020(2)
	Se2	2*e*	0.33750(9)	1/4	0.00500(5)	1	0.035(2)
	Se3	2*e*	0.72520(8)	1/4	0.58690(5)	1	0.031(2)
	Se4	2*e*	0.15290(9)	1/4	0.65990(5)	1	0.029(2)
	Se5	2*e*	0.01680(8)	1/4	0.13510(4)	1	0.022(2)
**5.9**	Nb	2*e*	0.60260(8)	1/4	0.86120(6)	1	0.044(3)
	Cr	2*e*	0.83970(13)	1/4	0.40240(10)	1	0.029(4)
	Se1	2*e*	0.27460(9)	1/4	0.25870(6)	1	0.029(3)
	Se2	2*e*	0.15620(9)	1/4	0.00200(7)	1	0.037(3)
	Se3	2*e*	0.36690(9)	1/4	0.57970(6)	1	0.039(3)
	Se4	2*e*	0.00770(9)	1/4	0.66730(7)	1	0.041(3)
	Se5	2*e*	0.62130(9)	1/4	0.13660(6)	1	0.043(3)
**14.6**	Nb	2*e*	0.60400(10)	1/4	0.86190(8)	1	0.054(3)
	Cr	2*e*	0.84030(15)	1/4	0.41260(12)	1	0.039(5)
	Se1	2*e*	0.26890(12)	1/4	0.26610(9)	1	0.060(4)
	Se2	2*e*	0.14470(10)	1/4	0.00140(9)	1	0.048(4)
	Se3	2*e*	0.36690(12)	1/4	0.57540(9)	1	0.066(5)
	Se4	2*e*	0.00440(12)	1/4	0.67490(10)	1	0.059(4)
	Se5	2*e*	0.62860(12)	1/4	0.13850(9)	1	0.049(3)

### High-Pressure Electrical Resistance Measurements

High-pressure electrical resistance measurements were conducted using a Be–Cu diamond anvil cell with 300 μm culet anvils in an in-house physical properties measurement system. A T301 stainless-steel gasket (200 μm thick) was preindented to 30 μm, and a 280 μm hole was drilled at its center. The gasket surface was insulated with a cubic boron nitride-epoxy mixture. Single-crystal CrNbSe_5_ samples were measured in a van der Pauw four-probe configuration using Pt foil electrodes. Two independent runs were performed on separate crystals with powdered NaCl as the pressure-transmitting medium. Pressure was applied at room temperature and calibrated by ruby fluorescence.^45^

### High-Pressure Raman Spectroscopy Measurements

High-pressure Raman measurements were performed using a diamond anvil cell with rhenium gaskets, a ruby pressure calibrant, and a 4:1 methanol–ethanol pressure medium. Raman spectra were collected at room temperature up to 40 GPa using a 532 nm laser with a constant power of 40 mW.

### Electronic Structure Calculations

Density functional theory (DFT) calculations were performed using Quantum ESPRESSO (v7.4.1).^46,47^ Projector-augmented wave (PAW) pseudopotentials from the PSlibrary were employed together with the Perdew–Burke–Ernzerhof (PBE) exchange-correlation functional.^48,49^ A kinetic-energy cutoff of 300 Ry was used for the wave functions, with the charge-density cutoff set to eight times this value. Brillouin-zone integrations were carried out using a 5 × 11 × 5 Monkhorst–Pack *k*-point mesh.^50^ Convergence tests ensured that the total energy varied by less than 1 meV per atom, and the self-consistent-field convergence threshold was set to 10^–9^ Ry. High-symmetry *k*-path generation for band-structure calculations was performed using the Spglib library.^51,52^ Crystal orbital Hamilton population (COHP) analyses were conducted using LOBSTER (v5.1.1) together with LOPOSTER.^53,54^ Fermi surfaces were visualized using FermiSurfer.^55^

## Results and Discussion

Low-dimensional solids are often described as stacks of slabs or juxtapositions of one-dimensional building units. CrNbSe_5_ is such a low-dimensional material, crystallizing in the monoclinic space group *P*2_1_/*m* at ambient pressure (see Figure 1a). Its structure can be described as (Cr_2_)_oct_(Nb_2_)_tp_Se_10_, consisting of chains of edge-sharing CrSe_6_ octahedra (oct) and NbSe_6_ trigonal prisms (tp), characteristic of the FeNb_3_Se_10_ structure type. This anisotropic connectivity renders the lattice particularly sensitive to external pressure, providing an effective route to tune both structure and physical properties.

**Figure 1:**
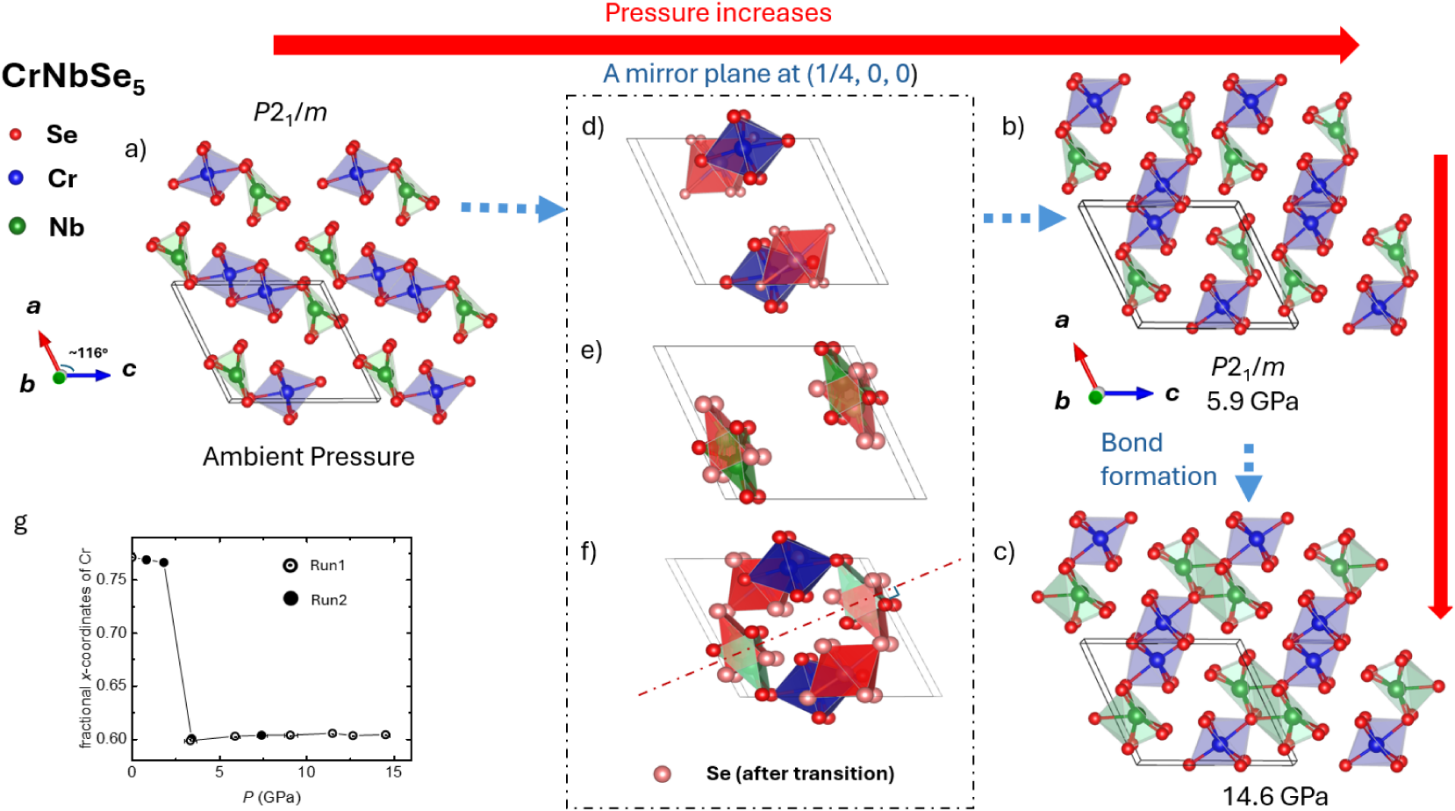
Crystal structure of CrNbSe_5_ and mirror-plane-mediated iso-symmetric structural transition under pressure. Panels (a)-(c) show the ambient- and high-pressure *P*2_1_/*m* crystal structures visualized using VESTA. The red arrow indicates the direction of increasing pressure. Panels (d)-(f) illustrate the pressure-induced reorganization of atomic positions associated with the iso-symmetric transition; red polyhedra highlight the coordination units undergoing positional rearrangement across the transition. The red polyhedra highlight the CrS6 environment following the transition. (g) The fractional *x*-coordinates of Cr. Solid symbols indicate the data from the sample 2 run 2.

To investigate the pressure response, high-pressure SCXRD measurements were performed up to 14.6 GPa. Selected refinement parameters of representative pressures are summarized in Table 1. Across the entire pressure range investigated, refinements in the monoclinic *P*2_1_/*m* space group consistently yielded the best agreement, with no evidence for symmetry lowering. Atomic coordinates refined at ambient pressure and representative pressures of 0.8, 5.9, and 14.6 GPa are listed in Table 2. Analysis of the refined structures reveals that the *y* and *z* atomic coordinates remain largely invariant with pressure, whereas significant changes in the *x* coordinates emerge above 5.9 GPa. As illustrated in Figure 1a-c, increasing pressure to 5.9 GPa induces a pronounced rearrangement of atomic positions while preserving the overall crystallographic symmetry, marking an iso-symmetric structural transition. This transition is reconstructive in nature, involving a reorganization of local coordination environments rather than a simple, continuous atomic displacement driven by lattice compression. Above 5.9 GPa, the high-pressure phase remains structurally stable up to 14.6 GPa. In this regime, the global framework of the structure is preserved, and further compression primarily manifests as continuous changes in interatomic distances. No additional symmetry breaking or abrupt atomic rearrangements are observed, indicating that the pressure-induced phase is robust once formed. The microscopic origin of the iso-symmetric transition is clarified by examining the evolution of local coordination polyhedra (Figure 1d-f). Both the CrSe_6_ octahedra and NbSe_6_ trigonal prisms undergo substantial reorientation and changes in polyhedral connectivity during the transition. Following this rearrangement, several Cr–Se and Nb–Se distances shift into typical bonding ranges, consistent with the formation of new pressure-stabilized interactions.

While structural refinements reveal a symmetry-preserving yet reconstructive atomic rearrangement, the macroscopic lattice response provides complementary insight into how the framework accommodates compression and signals the emergence of a pressure-induced phase. Figure 2a-d summarizes the evolution of the lattice parameters as a function of pressure. All lattice parameters decrease monotonically upon compression, with total reductions approaching ∼10% at 14.6 GPa, consistent with progressive densification of the low-dimensional framework. The pressure dependence of the unit-cell volume is shown in Figure 2e and is well described by a Birch–Murnaghan equation of state, indicating the absence of a first-order volume collapse across the measured pressure range. Despite this smooth volumetric compression, repeated measurements consistently reveal a subtle anomaly near ∼3 GPa, most prominently reflected in the β angle and *c*-axis parameter in Figure 2b and d, respectively. In this pressure range, shown in Figure 2e, the distinct high-pressure structure is shown. This high-pressure structure has an interesting feature which is an apparent mirror plane at (1/4, 0, 0). This mirror plane is not a symmetry element required by the *P*2_1_/*m* space group, but it is a structural feature associated with the pressure-driven atomic rearrangement. To compare the 0.8 and 3.4 GPa structures, we applied a rigid translation to align the Nb site and then matched the five Se sites one by one in the best possible way. With this comparison, the Nb sites overlap exactly and three Se sites agree within 0.3 Å. In contrast, the Cr site still differs by about 0.6 Å, and other two Se sites show much larger differences of 1.7 Å for Se1 and 2.0 Å for Se4. These results show that the two structures look similar after rigid alignment, but they are not the same at the atomic level. For this reason, they cannot be treated as the same structural model in the SCXRD solution process. High-pressure Raman measurements show anomalies in the same pressure range (Figure S1), which support this observation. Together, these results indicate a pressure-induced coordination rearrangement rather than a conventional symmetry-breaking phase transition.

**Figure 2:**
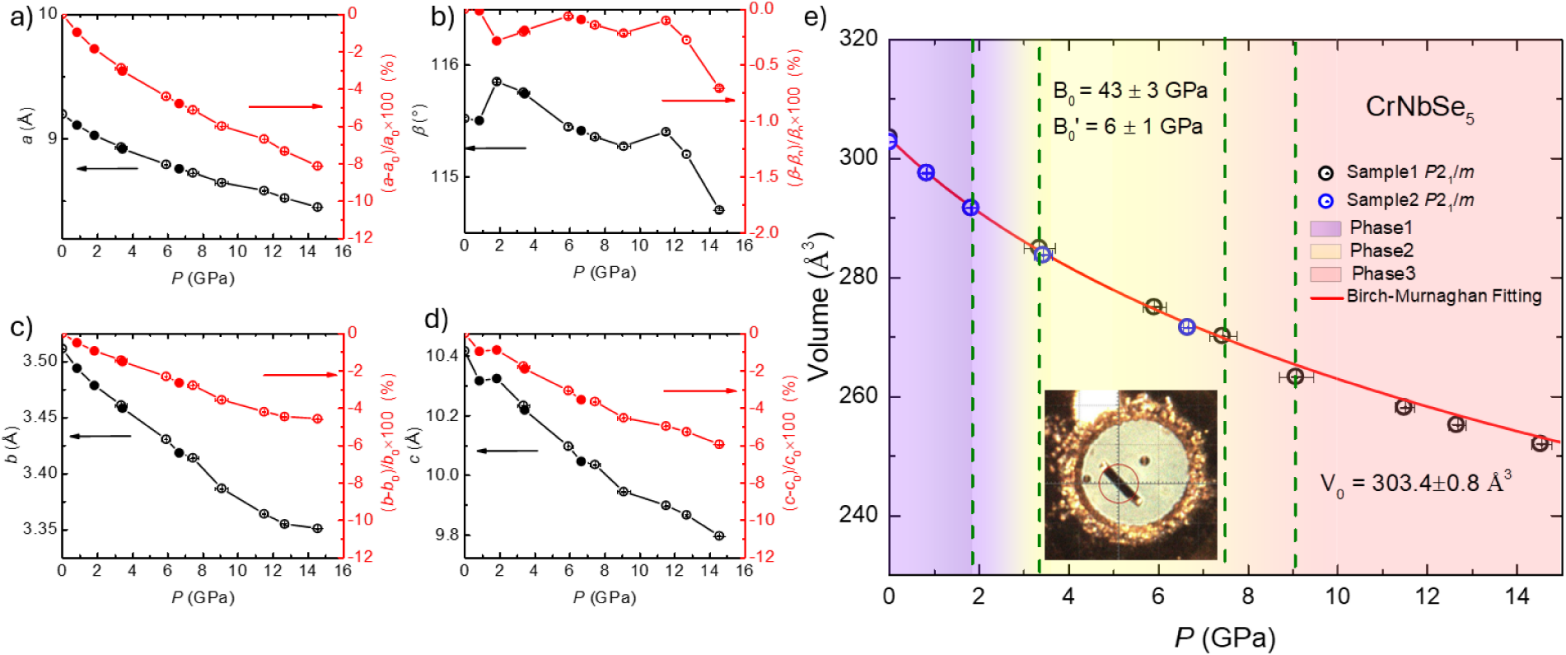
Pressure-dependent evolution of the lattice parameters and unit-cell volume of CrNbSe_5_. Panels (a)-(d) show the lattice parameters as a function of pressure; the red panels indicate the relative changes expressed as percentages. Solid symbols indicate the data from sample 2 run 2. (e) Unit-cell volume versus pressure with a Birch–Murnaghan equation-of-state fit. The inset displays the gasket and sample chamber at 5.9 GPa; the red circle marks a diameter of 0.1 mm. The light-violet region denotes phase I, corresponding to the ambient-pressure atomic configuration, while the yellow region indicates the pressure-induced phase II with reconstructed atomic positions. The red region indicates phase III with Fermi surface reconstruction.

Although no clear phase transition is evident from the smooth evolution of the unit-cell volume, the pressure dependence of specific Cr–Se and Nb–Se interatomic distances provides deeper insight into subtle structural rearrangements and their influence on electronic properties. Figure 3 summarizes the evolution of selected bond lengths as a function of pressure, with the green dashed region highlighting the pressure range associated with the iso-symmetric structural transition. Overall, the Cr–Se and Nb–Se bond lengths vary only modestly across the transition, indicating that the pressure-induced transformation primarily involves a reorganization of coordination polyhedra rather than simple atomic displacements. Repeated measurements show good reproducibility of most bond distances over the entire pressure range, with the notable exception of the Nb–Se bond (D2). The larger uncertainty and enhanced variability of D2 suggest that this interaction plays a key role in accommodating the polyhedral rearrangement near ∼3 GPa. In addition, a pronounced change in the D2 bond length is observed around ∼8 GPa, closely coinciding with anomalies detected in high-pressure electrical resistance measurements (Figure S2a), highlighting a strong coupling between local bonding reconstruction and electronic transport behavior. We suggest that D2 may form the bond like the collapsed tetragonal in 112 system after 8 GPa. Despite these pressure-induced structural and electronic anomalies, no superconductivity was detected up to 55 GPa, which may reflect differences in sample response or pressure conditions. As shown in Figure S2, resistance measurements reveal a pressure-driven evolution of the electronic state in CrNbSe_5_: the material transitions from a semiconducting state to a semimetallic state between ∼2.3 and 5 GPa, accompanied by a marked decrease in resistance. Upon further compression, CrNbSe_5_ reenters a semiconducting behavior at 10.7 GPa (The resistance increases with decreasing temperature.) after exhibiting semimetallic behavior at 7.5 GPa. Notably, the pressures associated with these semiconductor-semimetal–semiconductor transitions correlate closely with changes in the Nb–Se bonding interaction (D2), underscoring the central role of local chemical bonding in governing the reversible electronic phase evolution under pressure.

**Figure 3:**
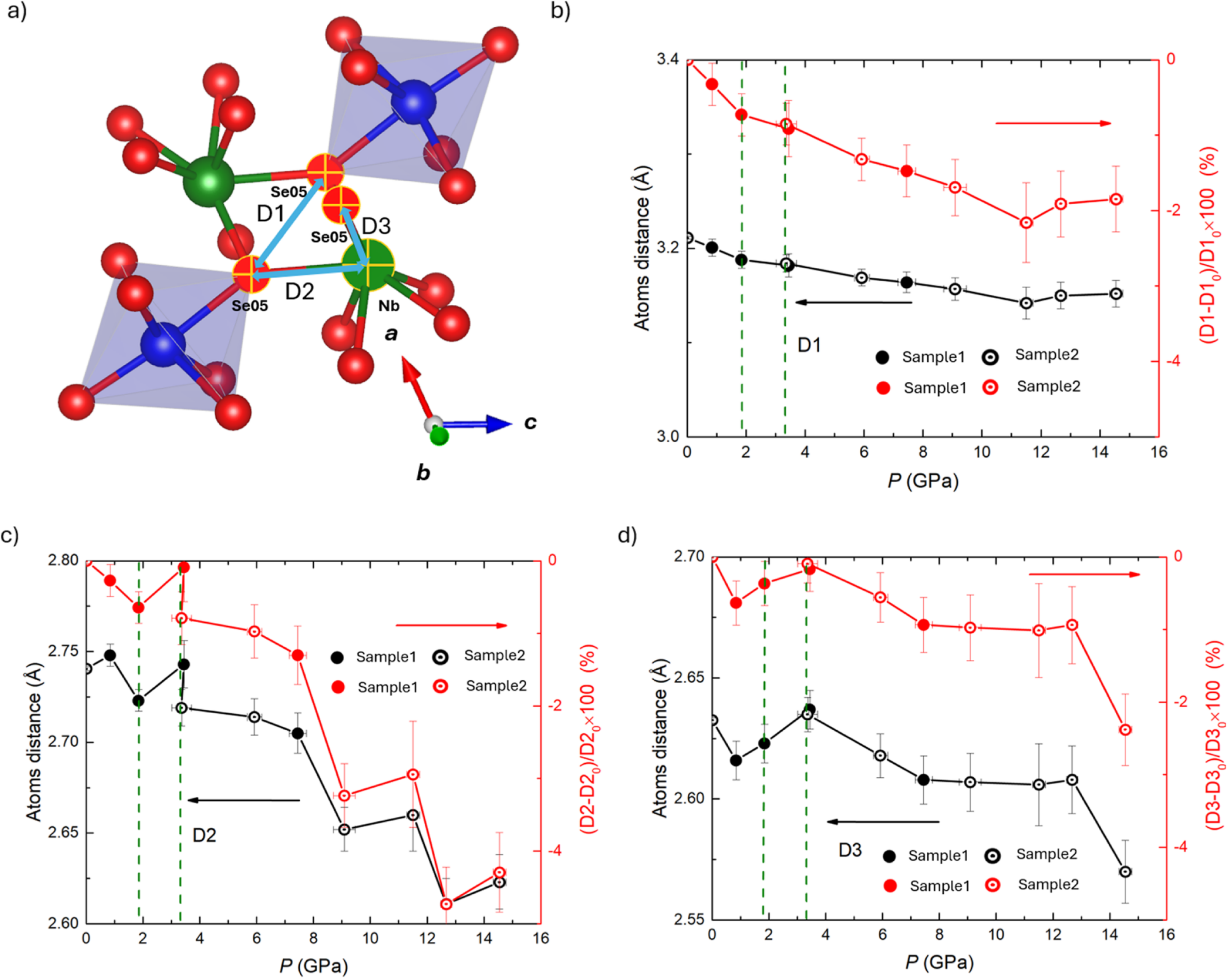
Pressure-dependent evolution of selected interatomic distances in CrNbSe_5_. (a) Schematic illustration of the local coordination environment highlighting the Se5 site (D1), which coordinates to two symmetry-related Nb atoms, giving rise to two distinct Nb–Se distances (D2 and D3). Panels (b)-(d) show the pressure dependence of these interatomic distances. The red panels indicate the relative changes in bond lengths, expressed as percentages. Solid symbols indicate the data from sample 2 run 2.

To elucidate the evolution of chemical bonding and its energetic consequences under pressure, electronic structure calculations were performed for CrNbSe_5_ at selected pressures. The calculated electronic density of states (DOS) is shown in Figure S3. At ambient pressure, CrNbSe_5_ exhibits a finite DOS at the Fermi level, indicating a metallic electronic structure. Upon compression, the overall DOS broadens, with spectral weight extending to lower energies below −6 eV and higher energies above +2 eV relative to the ambient-pressure case. This bandwidth expansion reflects enhanced orbital overlap induced by pressure. To gain insight into the pressure-dependent chemical bonding, crystal orbital Hamilton population (COHP) analyses and corresponding integrated COHP (−ICOHP) values were evaluated. Consistent with the structural motifs, the dominant orbital interactions arise from Cr–Se and Nb–Se bonds, as shown in Figure 4. Among these, the Nb–Se interaction associated with the D2 bond exhibits the most pronounced pressure sensitivity and is therefore expected to play a critical role in governing the electronic transport behavior. As shown in Figure 4a, the Nb–Se (D2) COHP curve places the Fermi level within a nonbonding to weakly antibonding region, indicating that small pressure-induced changes in bonding can strongly influence carrier behavior. Analysis of the −ICOHP values reveals a significant modification of the Nb–Se (D2) interaction between 7.4 and 9 GPa, in excellent agreement with both the observed structural rearrangement and anomalies in electrical resistance. This behavior can be rationalized within a bonding framework (*S*_*ij*_^2^/Δ*E* ^(0)^) in which pressure enhances orbital overlap (increasing the overlap integral *S*_*ij*_), while the energy separation between interacting states (Δ*E* ^(0)^) remains largely unchanged, leading to strengthened covalent interactions. To further assess the overall bonding evolution, COHP analyses for all relevant atomic pairs (Cr–Cr, Cr–Se, Nb–Nb, Nb–Se, and Se–Se) were performed up to 9 GPa (Figure S4). Notably, a distinct Cr–Nb interaction emerges above ∼7.4 GPa and becomes observed at 9 GPa, signaling the onset of new interchain or interpolyhedral coupling under compression. This emergent interaction provides additional support for a pressure-driven reorganization of the bonding network, rather than a simple lattice contraction, and highlights the key role of chemical bonding reconstruction in stabilizing the high-pressure electronic states of CrNbSe_5_.

**Figure 4:**
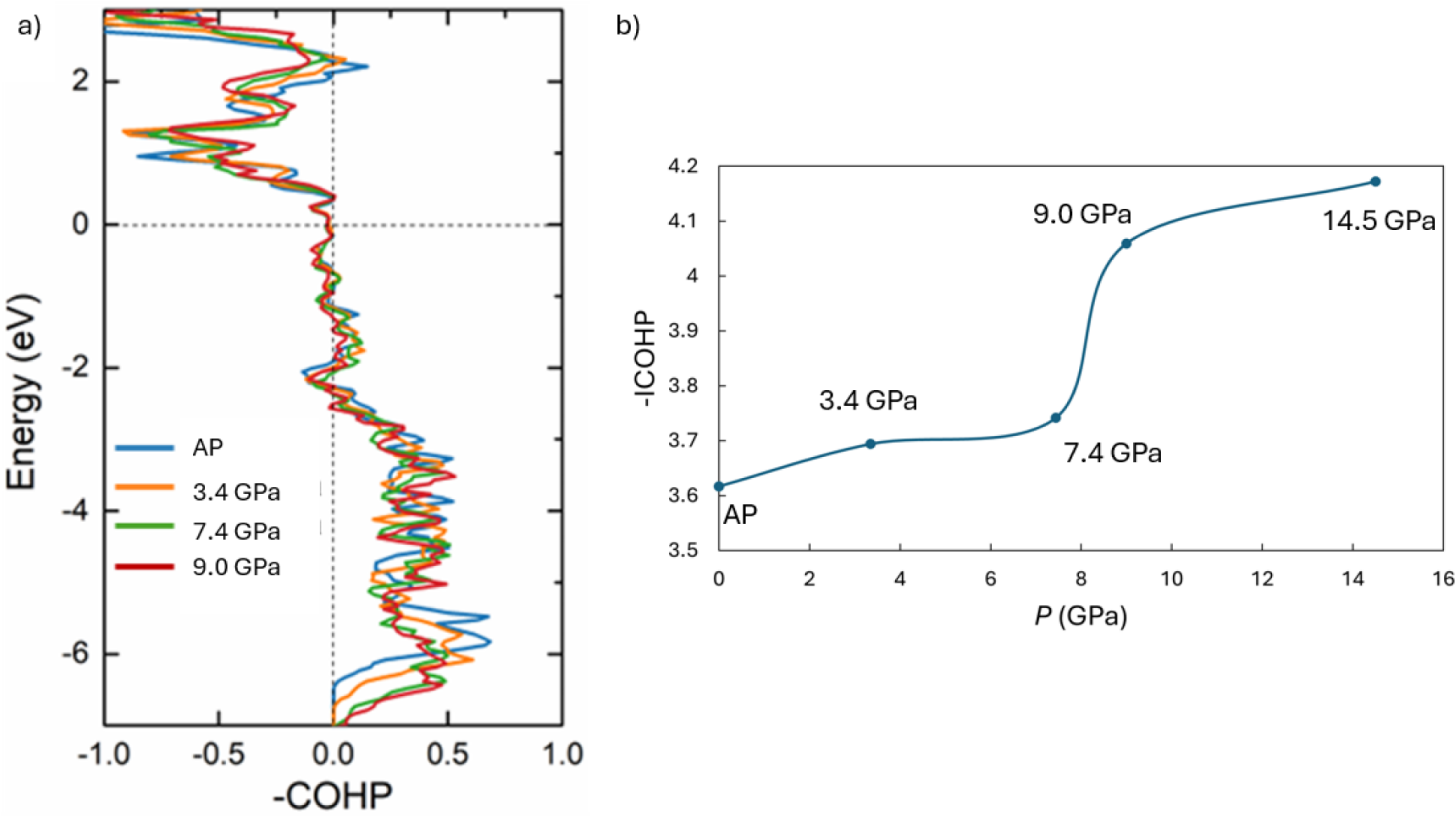
Pressure-dependent electronic structure of CrNbSe_5_. (a) Crystal orbital Hamilton population (COHP) curves for the Nb–Se (D2) bond at selected pressures up to 9 GPa. (b) Corresponding integrated COHP (−ICOHP) values for the Nb–Se (D2) interaction as a function of pressure, highlighting the pronounced change above 7.4 GPa.

Figure 5 gives the evolution of the Fermi surface cross-section of CrNbSe_5_ in the *k*_*x*_-*k*_*y*_ plane, *k*_*y*_-*k*_*z*_ (Figure S5), and 3D-Fermi surfaces (Figure S6) with the pressure range from ambient to 14.5 GPa. With increasing pressure, the topology of the Fermi surface evolves continuously at low pressures, while a pronounced reconstruction occurs around 8 GPa. In this pressure range, several Fermi surface sheets undergo neck formation and breaking, accompanied by the appearance/disappearance of small electron- and hole-like pockets. These features indicate a pressure-induced Lifshitz transition, i.e., a change in Fermi surface topology without crystallographic symmetry breaking, consistent with the anomalies observed in transport and bonding analyses. Remarkably, a relatively small pressure between Figure 5d and e already produces a change in the D2 bonding distance (Figure 3c), which in turn leads to a pronounced reconstruction of the Fermi surface. Given that other bonding distances exhibit only weak pressure dependence in this regime, the D2 interaction is identified as the key structural parameter governing the pressure-induced electronic reconstruction and the associated changes in the physical properties of CrNbSe_5_. At higher pressures, however, such as 14.5 GPa, the Fermi surface evolves into a more conventional metallic topology, and no superconducting signature is observed.

**Figure 5:**
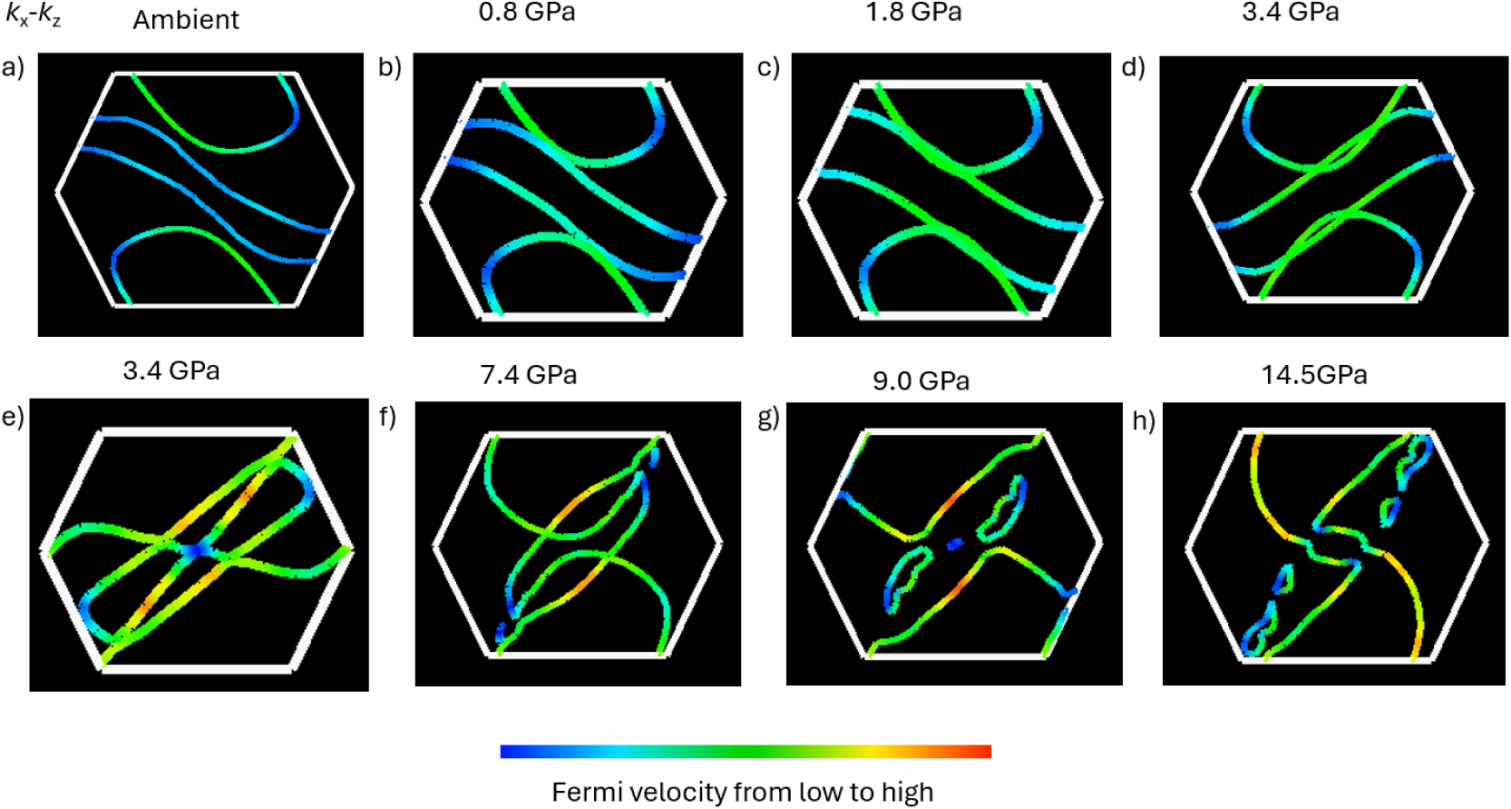
Pressure evolution of the Fermi surface cross-sections of CrNbSe_5_ in the *k_x_*-*k_y_* plane and *k_y_*-*k_z_* (Figure S4). Calculated Fermi surface at (a) ambient pressure, (b) 0.8 GPa, (c) 1.8 GPa, (d) and (e) 3.4 GPa, (f) 7.4 GPa, (g) 9.0 GPa, and (h) 14.5 GPa is shown within the first Brillouin zone (white hexagon). (d) and (e) come from the structures of two different measurements. One is around 3.35 GPa (d), and another is 3.43 GPa. Considering the precision of pressure measurement using ruby, we marked them as 3.4 GPa for both.

## Conclusion

In summary, applied pressure induces a fully reversible iso-symmetric structural transition in the quasi-one-dimensional compound CrNbSe_5_ around 3 GPa, accompanied by clear changes in electronic transport without a change in crystallographic symmetry. High-pressure X-ray diffraction, combined with Raman spectroscopy, transport measurements, and electronic structure calculations show that this transition arises from a cooperative reorganization of local Cr–Se and Nb–Se bonding environments and packing method rather than conventional symmetry breaking or dramatic volume collapse. Pressure continuously modifies coordination polyhedra and interchain connectivity, allowing reversible tuning between semiconducting and semimetallic regimes through direct modulation of chemical bonding around 8 GPa. In this pressure range, several Fermi surface sheets undergo neck formation and breaking, accompanied by the emergence and disappearance of small pockets, indicating a change in Fermi surface connectivity. Such a topological transformation of the Fermi surface occurring without any crystallographic symmetry change is characteristic of a Lifshitz transition. This pressure range coincides with pronounced anomalies in the Nb–Se bonding interaction (Figure 4) and the nonmonotonic evolution of electrical resistance (Figure S2), establishing a direct link between pressure-tuned chemical bonding, electronic topology, and electrical transport behavior in CrNibSe_5_. These results establish CrNbSe_5_ as a model system in which electronic phase switching is achieved through symmetry-preserving structural reconfiguration and suggest that pressure-controlled bond rearrangement provides a general route for tuning electronic states in low-dimensional chalcogenides.

## Supplementary Material



## Notes

Raman measurements of CrNbSe_5_; High-pressure resistance measurements of CrNbSe_5_; The calculated electronic density of states (DOS); Pressure evolution of COHP curves for all relevant atomic interactions in CrNbSe_5_; Pressure evolution of the Fermi surface cross-section of CrNbSe_5_ in the *k*_*y*_-*k*_z_; Pressure evolution of the 3D Fermi surface of CrNbSe_5_ (PDF)
