# Supplementary material for: Observation of Iso-Symmetric Structural and Lifshitz Transitions in Quasi-One-Dimensional CrNbSe5
Source: J Phys Chem C Nanomater Interfaces. 2026 Apr 30;130(19):6821–30. doi: 10.1021/acs.jpcc.6c01534 (PMC13181763; doi:10.1021/acs.jpcc.6c01534)

Supplementary Information

Observation of Iso-Symmetric Structural and Lifshitz Transitions in Quasi-one-dimensional $CrNbSe_5$

Mingyu Xu[1], Cheng Peng[1], Shuyuan Huyan[2,3], Wenli Bi[4], Su-Yang Xu[5], Sergey L. Bud'ko[2,3], Paul C. Canfield[2,3], Weiwei Xie[1*]

1. Department of Chemistry, Michigan State University, East Lansing, MI 48824, USA
2. Ames National Laboratory, Ames, IA 50011, USA
3. Department of Physics and Astronomy, Iowa State University, Ames, IA 50011, USA
4. SmartState Center for Experimental Nanoscale Physics, Department of Physics and Astronomy, University of South Carolina, Columbia, SC 29208, USA
5. Department of Chemistry and Chemical Biology, Harvard University, Cambridge, MA 02138, USA

*Corresponding author: Dr. Weiwei Xie (xieweiwe@msu.edu)

Table of Contents

Fig. S1 Raman measurements of $CrNbSe_5$.……………………………………………..…...……S2

Fig. S2 High-pressure resistance measurements of $CrNbSe_5$.……………………………….…….S3

Fig. S3 The calculated electronic density of states (DOS).……………………………….…….S4

Fig. S4 Pressure evolution of COHP curves for all relevant atomic interactions in $CrNbSe_5$….S5

Fig. S5 Pressure evolution of the Fermi surface cross section of $CrNbSe_5$ in the k_y-k_z ……….…….S6

Fig. S6 Pressure evolution of the 3D Fermi surface of $CrNbSe_5$ ……………………………..……S7

Figure 1. (*a*) Raman measurements of $CrNbSe_5$ around the pressure reading. (*b*) Raman shift as a function of pressure.

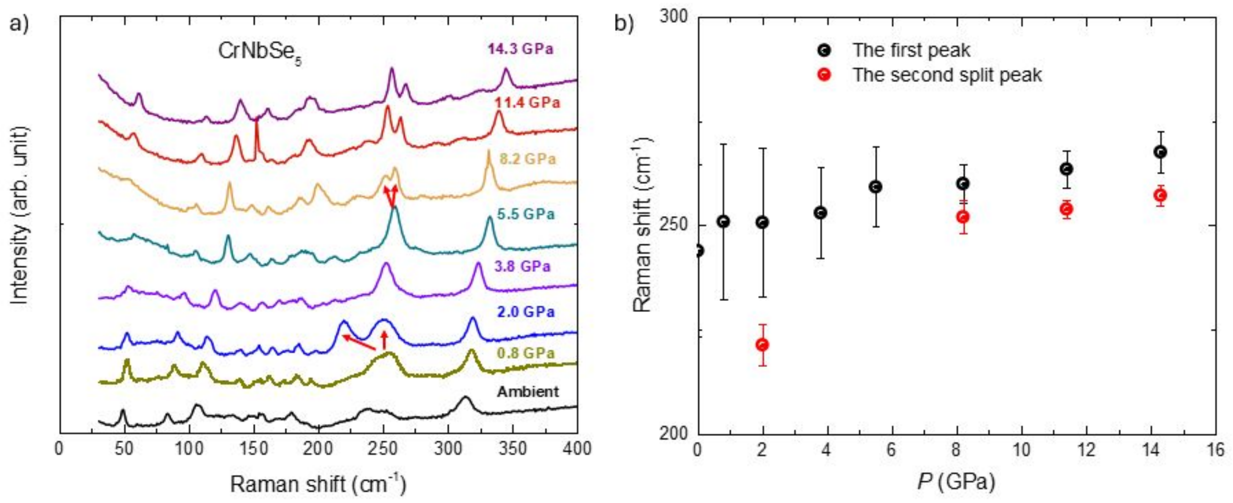

Figure S2. High-pressure resistance of $CrNbSe_5$. Blue dashed lines indicate the transitions around 3 and 8 GPa.

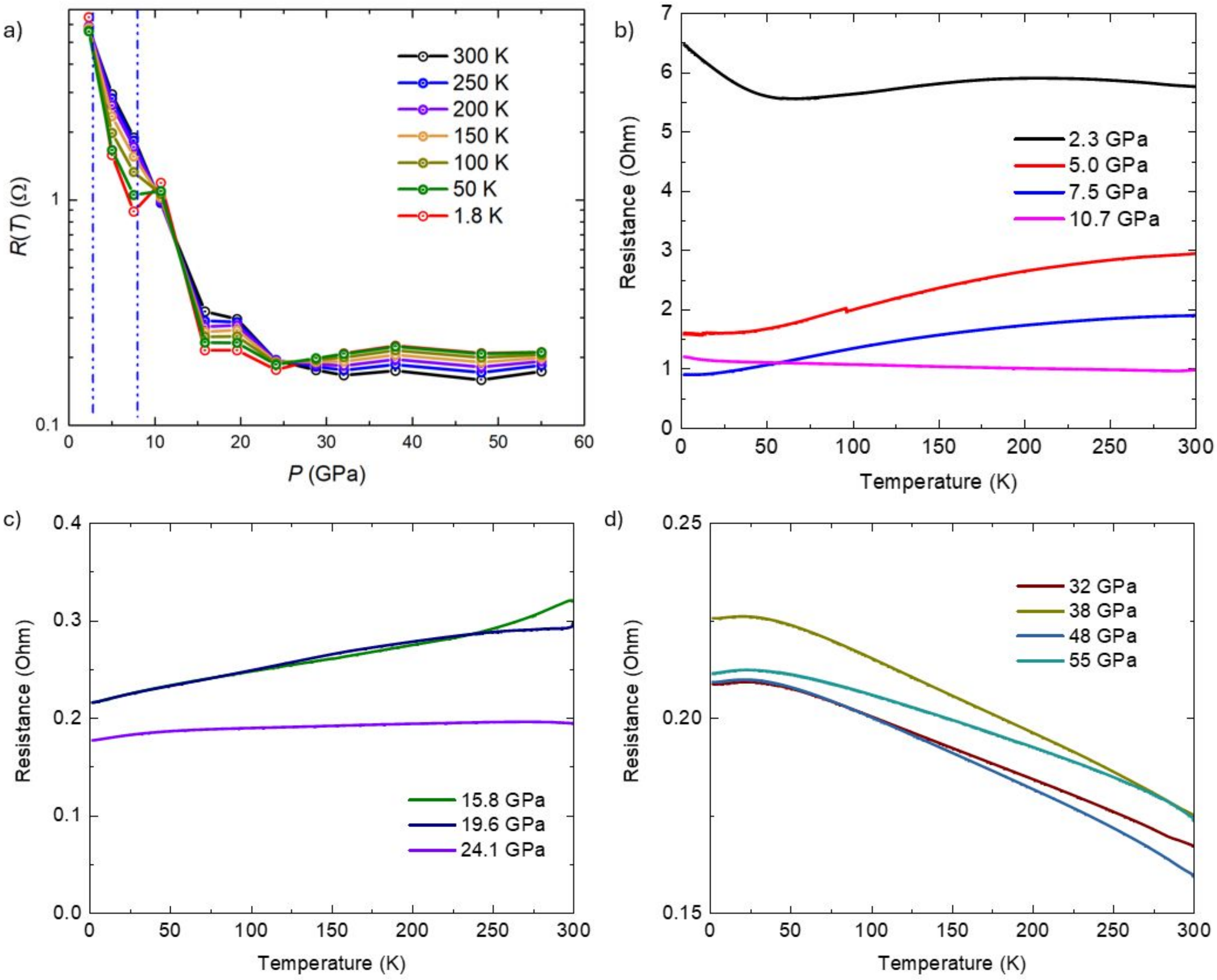

Figure S3. The calculated electronic density of states (DOS).

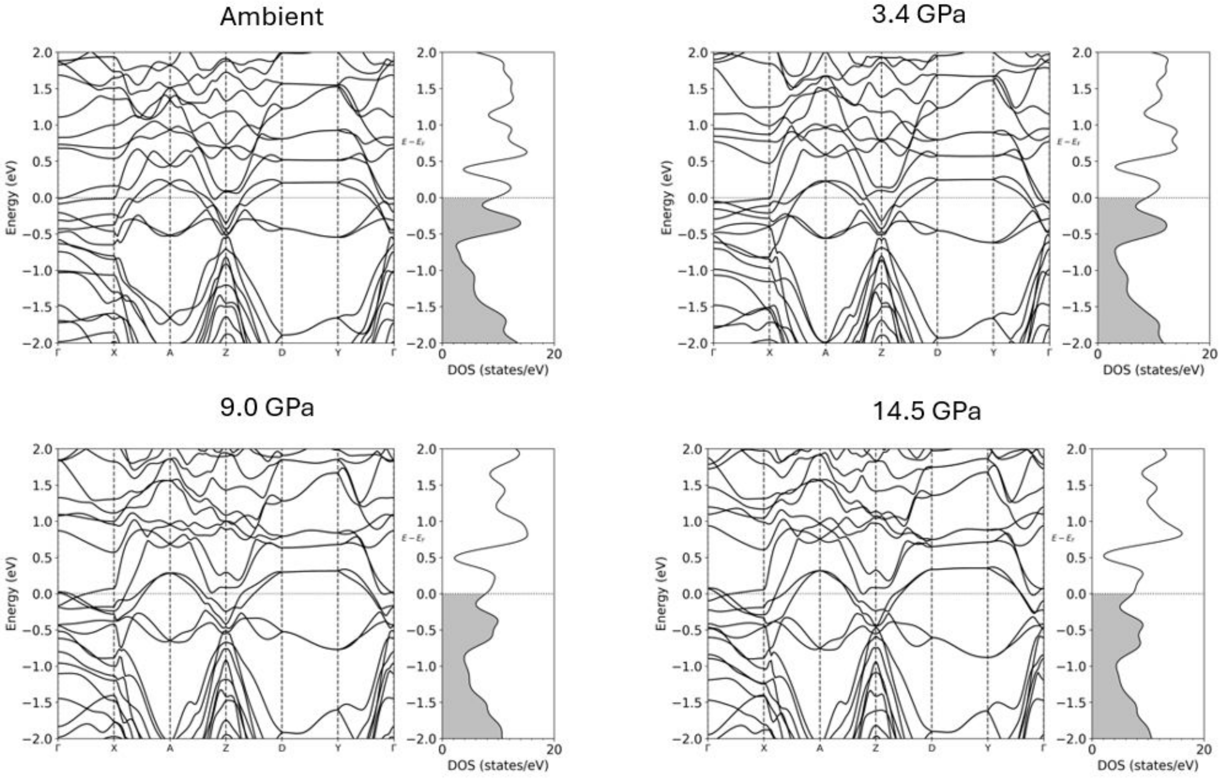

Figure S4. Pressure evolution of crystal orbital Hamilton population (COHP) curves for all relevant atomic interactions in $CrNbSe_5$, including Cr-Cr, Cr-Se, Nb-Nb, Nb-Se, and Se-Se. Panels show results at (*a*) ambient pressure, (*b*) 3.4 GPa, (*c*) 7.4 GPa, and (*d*) 9.0 GPa. Notably, a Cr-Nb interaction emerges at high pressure, becoming evident at 9 GPa.

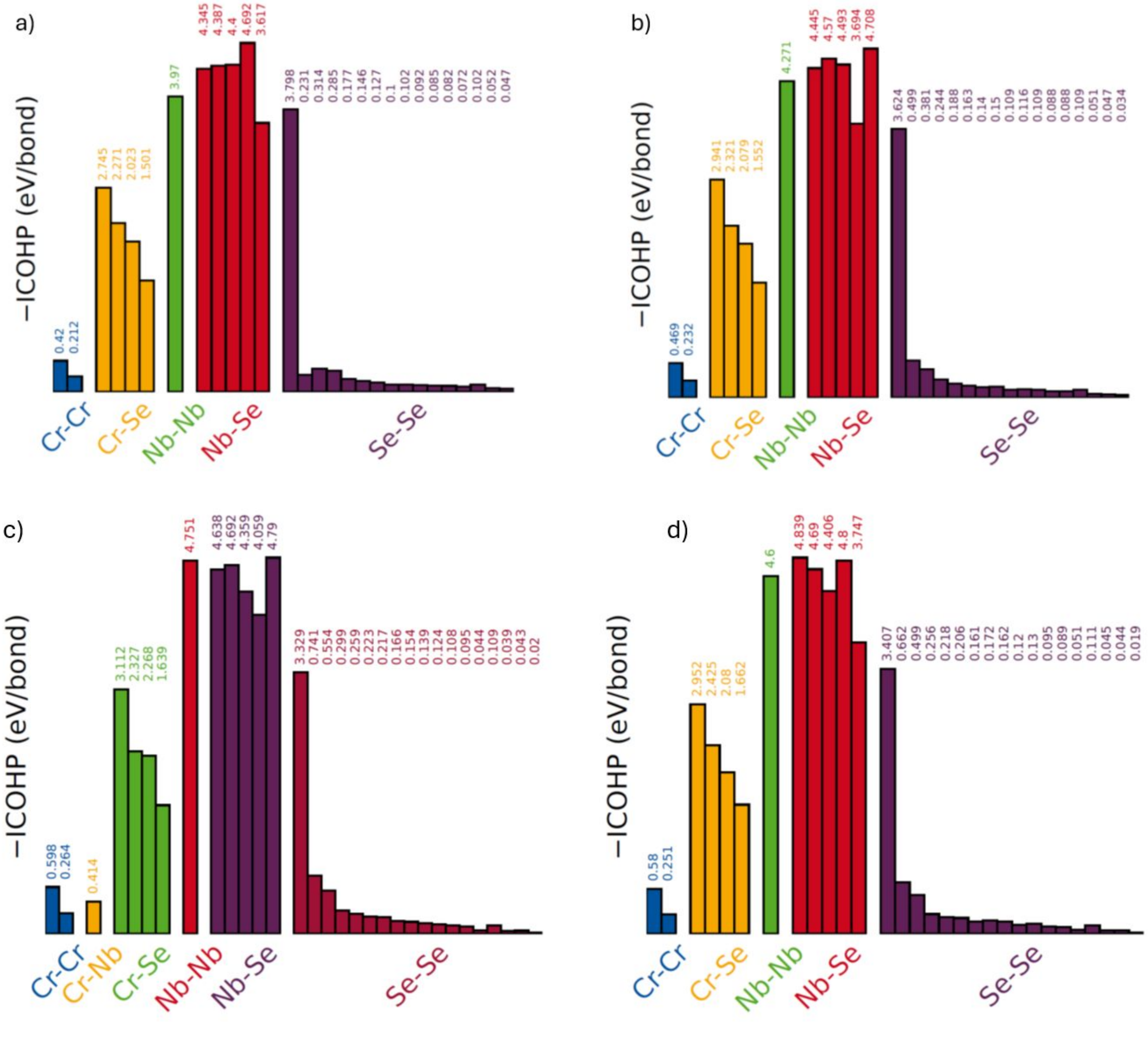

Figure S5 Pressure evolution of the Fermi surface cross section of $CrNbSe_5$ in the k_y-k_z .

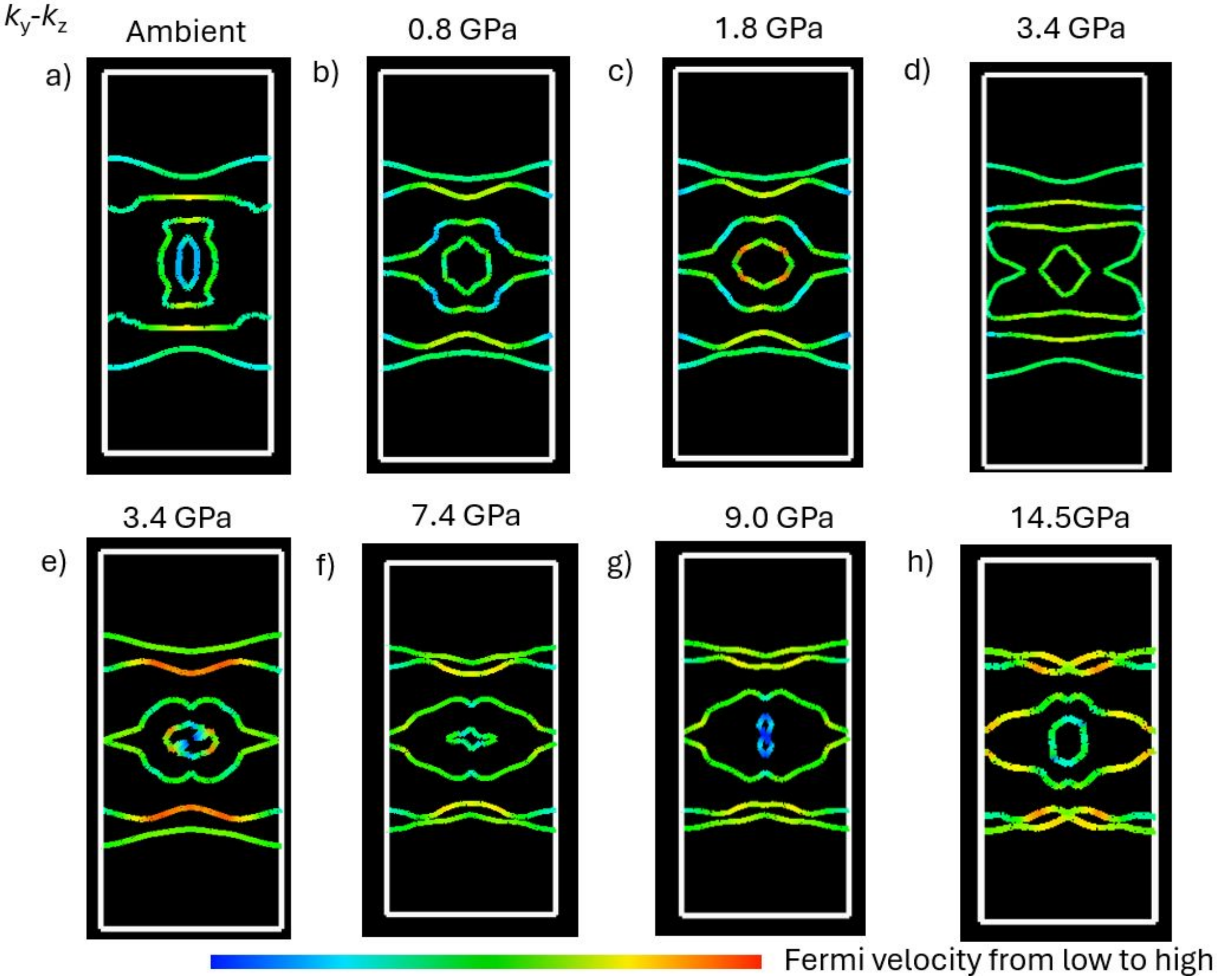

Figure S6 Pressure evolution of the Fermi surface cross section of $CrNbSe_5$.

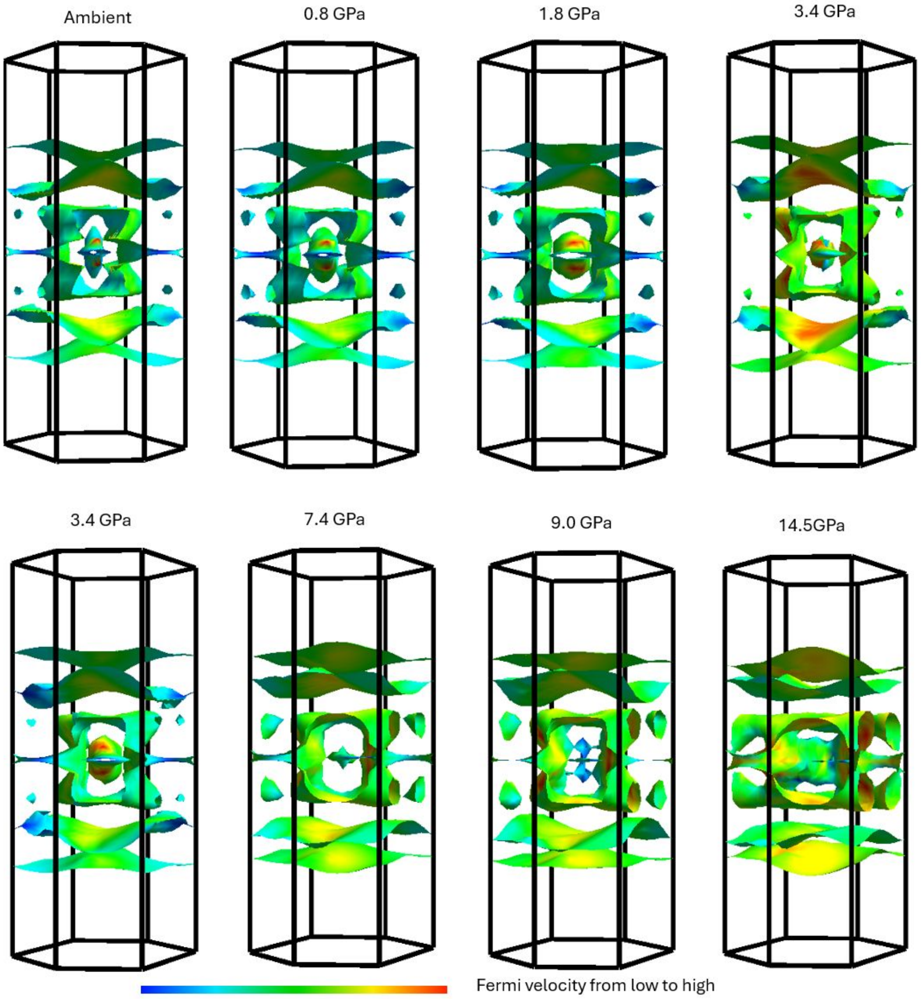

Supplement: Supplementary file 1 [file jp6c01534_si_001.pdf]
